# 1751. Evaluation of Antimicrobial Stewardship Interventions in Primary Care Clinics

**DOI:** 10.1093/ofid/ofac492.1381

**Published:** 2022-12-15

**Authors:** Tina Zheng, Suri Mayer, Samantha Cham, Patrizia Niceforo, Monica Ghitan

**Affiliations:** New York-Presbyterian Hospital, Queens, New York; Maimonides Medical Center, Brooklyn, New York; Maimonides Medical Center, Brooklyn, New York; Maimonides Medical Center, Brooklyn, New York; Maimonides Medical Center, Brooklyn, New York

## Abstract

**Background:**

The goal of antimicrobial stewardship is to optimize antimicrobial therapy and clinical outcomes while minimizing the unintended consequences of antimicrobial use. With the majority of antibiotics prescribed in the outpatient setting, optimizing antimicrobial prescribing across the care continuum is necessary. We aimed to evaluate the effect of antimicrobial stewardship interventions on the rate of inappropriate antibiotic prescribing in three adult primary care clinics affiliated with a tertiary care hospital.

**Methods:**

Antibiotic prescriptions were identified by filtering common antibiotics prescribed prior to and post intervention. Prescriptions were excluded if they were prescribed pre-procedure or if the patient had a recent hospitalization within 30 days of the outpatient visit. Appropriateness of antibiotics was determined by indication, agent, dose and duration. Institutional guidelines were created for most common infections treated in the pre-intervention period. Education to outpatient providers was completed in two in-person visits.

**Results:**

A total of 258 prescriptions were reviewed between the pre- and post-intervention periods. The majority of patients were female (73-76%) and the median age was similar between both cohorts. The most common infection treated during the pre-intervention period was urinary tract infection, whereas skin and soft tissue infection was most common in the post-intervention period. During the baseline period, the inappropriate prescribing rate was 61%. After intervention, the rate was reduced to 55%.

**Conclusion:**

The use of outpatient antimicrobial stewardship guidelines and provider education to reinforce knowledge demonstrated reductions in inappropriate antibiotic prescribing.

**Disclosures:**

**All Authors**: No reported disclosures.

